# Echocardiographic and Clinical Outcomes of Concomitant Secondary Chordal Cutting to Surgical Myectomy in Hypertrophic Obstructive Cardiomyopathy: A Systematic Review and Meta-analysis

**DOI:** 10.1055/a-2434-7627

**Published:** 2024-11-18

**Authors:** Tijn Julian Pieter Heeringa, Romy R.M.J.J. Hegeman, Len van Houwelingen, Marieke Hoogewerf, David Stecher, Johannes C. Kelder, Pim van der Harst, Martin J. Swaans, Mostafa M. Mokhles, Ilonca Vaartjes, Patrick Klein, Niels P. van der Kaaij

**Affiliations:** 1 Department of Cardiothoracic Surgery, University Medical Centre Utrecht, Utrecht, The Netherlands; 2 Department of Cardiothoracic Surgery, St Antonius Hospital, Nieuwegein, Utrecht, The Netherlands; 3 Department of Cardiothoracic Surgery, Medical Centre Leeuwarden, Leeuwarden, Fryslân, The Netherlands; 4 Department of Clinical Epidemiology, St Antonius Hospital, Nieuwegein, Utrecht, The Netherlands; 5 Department of Cardiology, University Medical Centre Utrecht, Utrecht, Utrecht, The Netherlands; 6 Department of Cardiology, St Antonius Hospital, Nieuwegein, Utrecht, The Netherlands; 7 Department of Cardiovascular Epidemiology, Julius Center for Health Sciences and Primary Care, Utrecht, Utrecht, The Netherlands; 8 Department of Cardiothoracic Surgery, Amsterdam University Medical Centres, Amsterdam, Noord-Holland, The Netherlands; 9 Department of Cardiothoracic Surgery, Erasmus Medical Centre, Rotterdam, Zuid-Holland, The Netherlands

**Keywords:** cardiomyopathy, echocardiography (all modalities applications), heart valve surgery, mitral valve surgery

## Abstract

In patients who underwent surgical myectomy for hypertrophic obstructive cardiomyopathy (HOCM), additional mitral valve repair may offer additional benefits in terms of further reducing left ventricular outflow tract (LVOT) gradients, systolic anterior motion (SAM), and mitral regurgitation (MR). We performed a systematic review of the literature to evaluate the evidence of surgical myectomy with additional secondary chordal cutting in patients with HOCM. A systematic literature search in MEDLINE and EMBASE was performed until April 2024. The primary outcome studied was postoperative echocardiographic LVOT gradient. A random effects meta-analysis of means was performed for the primary outcome. The secondary outcomes studied were postoperative residual MR grade, 30-day new permanent pacemaker implantation, and in-hospital mortality. From 1,911 unique publications, a total of 6 articles fulfilled the inclusion criteria and comprised 471 patients with a pooled mean preoperative resting LVOT gradient of 84 mm Hg (95% confidence interval [CI]: 76–91). The postoperative pooled mean LVOT gradient was 11 mm Hg (95% CI: 10–12) with a low heterogeneity ( ***I*** ^2^ = 44%). The residual LVOT gradient exceeding 30 mm Hg was present in nine (1%) patients. MR grade 3 or 4 at hospital discharge was present in seven (1%) patients. The 30-day new permanent pacemaker implantation rate was 7% and the in-hospital mortality was 0.4%. This systematic review and meta-analysis demonstrate that combining surgical myectomy with secondary chordal cutting can be performed safely and effectively eliminate LVOT obstruction in HOCM patients. Further studies are needed to determine the additive effectiveness of additional secondary chordal cuttings.

## Introduction

Hypertrophic cardiomyopathy (HCM) is the most common inherited heart disease, with an estimated prevalence of up to 0.2% in the general population. 1 2 It is defined by increased left ventricular wall thickness (≥ 15 mm) not solely attributable to abnormal loading conditions and associated with sudden cardiac death, heart failure, and arrhythmias. 1 HCM often involves dynamic left ventricular outflow tract obstruction (LVOTO). Approximately 70% of HCM patients exhibit LVOTO, predominantly due to proximal septal hypertrophy and systolic anterior motion (SAM) of the anterior leaflet of the mitral valve. 3 Surgical myectomy remains the most commonly performed treatment for LVOTO and has been proven to abolish or substantially reduce LVOT gradients in over 90% of cases. Since hypertrophic obstructive cardiomyopathy (HOCM) patients might have mitral valve abnormalities (e.g., elongated mitral valve leaflets or abnormal papillary muscle insertion) that predispose them to SAM, 4 5 and SAM-related mitral regurgitation (MR), 6 7 additional mitral valve repair seems a reasonable and pathophysiologic oriented strategy to perform in addition to surgical myectomy. Although MR can often be eliminated without adjunctive mitral valve interventions in most HOCM cases, the 2023 European Society of Cardiology (ESC) Guidelines for the management of cardiomyopathies underscore that when moderate to severe MR cannot be corrected by surgical myectomy alone, mitral valve repair should be considered as an addition to surgical myectomy (class IIa, level of evidence C). 1

In the past decade, a wide range of mitral valve repair techniques have been described, including anterior mitral leaflet extension, anterior mitral leaflet retention, surgical edge-to-edge repair, and secondary chordal cutting. 8 9 10 11 Secondary chordal cutting in addition to surgical myectomy was first proposed by Ferrazzi et al who reported excellent outcomes. 12 Surgically targeting the thickened or fibrotic secondary chordae tendineae by cutting them is a strategy performed in various experienced centers, although current evidence mainly consists of multiple case series and cohort studies, and to date, a pooled outcome analysis is not yet available.

We performed a systematic review and meta-analysis to summarize the current evidence on the echocardiographic and clinical outcomes of HOCM patients undergoing surgical myectomy with additional secondary chordal cutting.

## Material and Methods

### Ethical Statement

Given the nature of our study, an institutional research board approval or informed written consent for publication was not required. This study complies with the recommendation of the Preferred Reporting Items for Systematic Reviews and Meta-Analyses (PRISMA) Extension statement, following the guidelines for reporting systematic reviews and meta-analysis. 13

### Protocol and Registration

The protocol of this systematic review and meta-analysis was prospectively registered with PROSPERO (ID = CRD42022303245).

### Secondary Chordal Cutting Mechanism

Secondary chordae of the mitral valve, also known as “strut” chordae, are defined as those that insert beyond the free edge and rough zone of the anterior mitral valve into the mid-body of the leaflet. 14 In HCM patients, these chordae may be abnormal (fibrotic, thickened, or shortened), leading to abnormal leaflet motion by tethering the anterior mitral valve leaflet anteriorly toward the left ventricular outflow tract (LVOT). This abnormal leaflet motion can contribute to SAM, MR, and LVOTO. These abnormal chordae could be removed (secondary chordal cutting) to improve the tenting area, thereby further reducing SAM, MR, and LVOTO.

### Literature Search Strategy

**Fig. 1:**
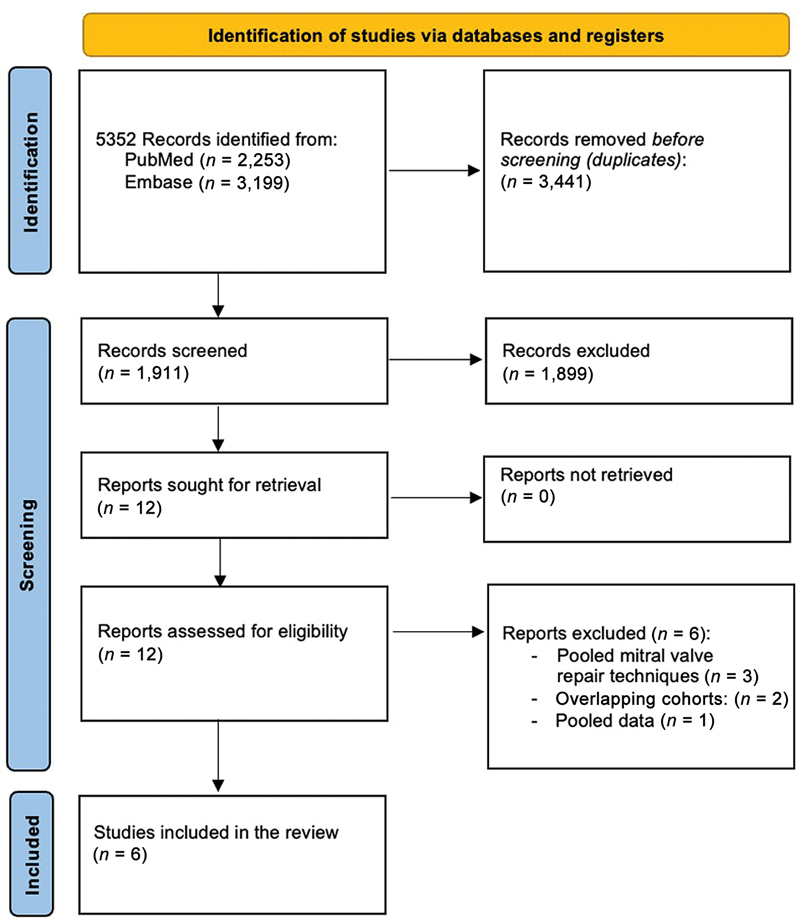
Preferred Reporting Items for Systematic Reviews and Meta-Analyses (PRISMA) flowchart demonstrating a selection of articles reporting on outcomes of secondary chordal cuttings in combination with surgical myectomy in patients with symptomatic hypertrophic obstructive cardiomyopathy.

The electronic databases MEDLINE (PubMed) and EMBASE were systematically searched up to April 12, 2024, to identify all mitral valve repair techniques, specifically secondary chordal cutting, in addition to surgical myectomy in HOCM patients. A clinical librarian was consulted for the design of the systematic search. The exact details for all searches are provided in the Supplementary Material ( search strings S1 , available in the online version only). Predefined criteria for selection were used to assess all articles. The article was written in accordance with PRISMA recommendations. 15 Fig. 1 shows a schematic representation illustrating an outline of the output observed at the distinct phases of the study's selection process. Two investigators (T.J.P.H. and R.R.M.J.J.H.) independently assessed the search results for compliance with the selection criteria at the title and abstract levels. Consensus was sought in case of differences between reviewers. Subsequently, potentially relevant articles were retrieved in full-text and were evaluated independently by the same investigators.

### Selection Criteria and Data Extraction

Articles that pooled patient data regarding different mitral valve and subvalvular mitral apparatus procedures, prohibiting separate analyses of the outcome of secondary chordal cutting, were excluded. Specifically, articles that pooled the results of patients who underwent secondary chordal cutting and patients who underwent mitral valve replacement were excluded, as were those articles that pooled the outcome of different mitral repair techniques. Further information on selection criteria could be found in Supplementary Methods S2 (available in the online version only). The primary outcome studied was postoperative resting LVOT gradient. The resting LVOT gradient was measured as a continuous variable and dichotomized into less than 30 and ≥30 mm Hg measured within 30 days postoperatively. The secondary outcome studied included the presence of postoperative MR. Postoperative MR measurement was defined as an echocardiographic measurement during hospital admission or in the outpatient clinic within 30 days after surgery. In this study, we indicated grades 3 and 4 as important residual MR. The occurrence of new permanent pacemaker implantation until 30 days and death was represented as a dichotomous variable (yes/no). Mortality was defined within two time frames (i.e., from the procedure until 30 days and from the procedure until 1 year). Further information on data extraction can be found in Supplementary Methods S2 (available in the online version only).

#### Critical Appraisal

All the articles that were included in the final analysis were evaluated for potential bias by two reviewers (L.v.H. and T.J.P.H.), according to the Risk of Bias in Non-Randomized Studies of Interventions (ROBINS-I) instrument bias assessment tool or the revised Cochrane risk-of-bias tool for randomized trials (RoB 2), depending on whether the study design was a retrospective cohort or a randomized controlled trial (RCT). The ROBINS-I offers a comprehensive assessment framework encompassing seven domains to evaluate the risk of bias in nonrandomized intervention studies. On the other hand, the RoB 2 tool provides a structured approach, comprising five domains, to evaluate the potential introduction of bias in the outcomes of various types of randomized trials. An assessment regarding feasible bias risk within each domain was deduced from gathered data and was categorized as either “high risk” or “low risk.” In instances of inadequate data, bias risk was categorized as “unclear.” In both the screening and bias stages, a pursuit of consensus was undertaken when disparities emerged between reviewers. Where disagreements occurred, a third reviewer was consulted to resolve the disagreement.

### Statistical Analysis

Continuous data were consolidated utilizing the “metamean” function in R for continuous variables and are reported as mean (95% confidence interval [CI]). This function is an integral component of the “meta” R package. 16 We performed a meta-analysis to assess the primary outcome (postoperative resting LVOT gradient). The primary outcome was also dichotomized into less than 30 and greater than 30 mm Hg. The secondary outcomes were reported as the number of observations (proportion). 17 Since there is no accepted scientific agreement on when and why to use random or common effect measures, we report both the random and common effects. Statistical heterogeneity was evaluated through chi-squared ( *χ* ^2^ ), *I* ^2^ tests, and *τ* ^2^ . The *I* ^2^ values of 0 to 49, 50 to 74, and 75 to 100% presented low, moderate, and high heterogeneity. The moderate and high heterogeneity outcomes were excluded from the meta-analysis. A *p* -value of less than 0.05 was considered statistically significant. Publication bias was assessed through visual inspection of funnel plots. All analyses were completed with R Statistical Software (version 4.1.1, Foundation for Statistical Computing, Vienna, Austria). All analyses were reviewed in collaboration with Cochrane Netherlands.

## Results

### Study Characteristics

The database queries retrieved 2,253 records from PubMed and 3,199 from EMBASE. After removing duplicates, the screening process involved 1,911 articles evaluated by the two reviewers. Of these, 1,899 were excluded based on titles and abstracts, predominantly since other procedures or mitral valve procedures other than secondary chordal cutting were investigated. Following the comprehensive evaluation of 12 full-text articles ( Fig. 1 ), the final selection consisted of 6 articles with a total of 471 patients. Six articles were excluded for different reasons **(** Supplementary Table S1 , available in the online version only). Four studies were designed as observational cohort studies, while two studies were designed as RCT. One study by Zyrianov et al stratified participants into two subgroups based on a septal thickness of ≤19 and ≥20 mm. The stratified subgroups are presented separately in Table 1 . 17 Within the majority of articles, the statistical analysis encompassed descriptive summaries and assessments of preoperative versus postoperative differences employing *t* -tests, chi-squared test, or Fisher's exact test. The study characteristics of the final selected articles are summarized in Table 1 .

**Table 1:** Study characteristics and baseline data

Study	*N*	Mitral valve repair technique	Method	Period	Age (y)	Gender (male)	AF	RBBB	NYHA class III–IV	LVEF	SAM	IVSd (mm)	Resting LVOT pressure gradient (mm Hg)	Provocative LVOT pressure gradient (mm Hg)	MR grades 0 and 1	MR grade 2	MR grade 3 and 4
**Russia**																	
Afanasyev et al 21	24	SCC	RCT	2016–2016	54 (12)	14 (58)	0	–	24 (100)	72 (8)	24 (100)	26 (23–29)	86 (26)	–	0	0	24 (100)
Bogachev-Prokophiev et al 20	40	SCC	RCT	2015–2016	50 (14)	14 (35)	0	–	27 (68)	76 (8)	40 (100)	27 (5)	92 (17)	–	0	0	40 (100)
**Romania**																	
Dorobantu et al 18	83	SCC	CS	2015–2020	52 (14)	51 (61)	26 (31)	–	49 (59)	63 (5)	–	24 (6)	93 (33)	–	12 (14)	39 (47)	32 (39)
**Italy**																	
Ferrazzi et al 12	39	SCC	CS	2011–2013	58 (13)	–	13 (33)	–	32 (82)	68 (6)	–	17 (1)	82 (43)	–	17 (44)	13 (33)	9 (23)
Zyrianov et al (≤19 mm IVSd) 17	64	SCC	CS	2015–2018	56 (12)	30 (47)	49 (77)	–	54 (84)	67 (6)	–	18 (1)	71 (41)	–	20 (32)	25 (39)	19 (30)
Zyrianov et al (≥20 mm IVSd) 17	161	SCC	CS	2015–2018	52 (15)	97 (60)	133 (82)	–	123 (76)	65 (6)	–	25 (4)	70 (34)	–	44 (27)	72 (45)	46 (28)
**Israel**																	
Ram et al 19	60	SCC	CS	2015–2020	61 (13)	30 (50)	20 (33)	–	44 (73)	65 (61–70)	–	25 (6)	91 (39)	–	–	–	30 (50)

Abbreviations: AF, atrial fibrillation; CS, cohort study; IVSd, interventricular septum thickness in diastole; LVEF, left ventricle ejection fraction; LVOT, left ventricular outflow tract; MR, mitral regurgitation; *N* , number of patients; NYHA, New York Heart Association; RCT, randomized controlled trial; RBBB, right bundle branch block; SAM, systolic anterior motion; SCC, secondary chordal cutting; –, data not reported.

Note: Values are mean ± standard deviation (SD), median (interquartile range), or *n* (%).

### Quality of Evidence and Bias Assessment

**Fig. 2:**
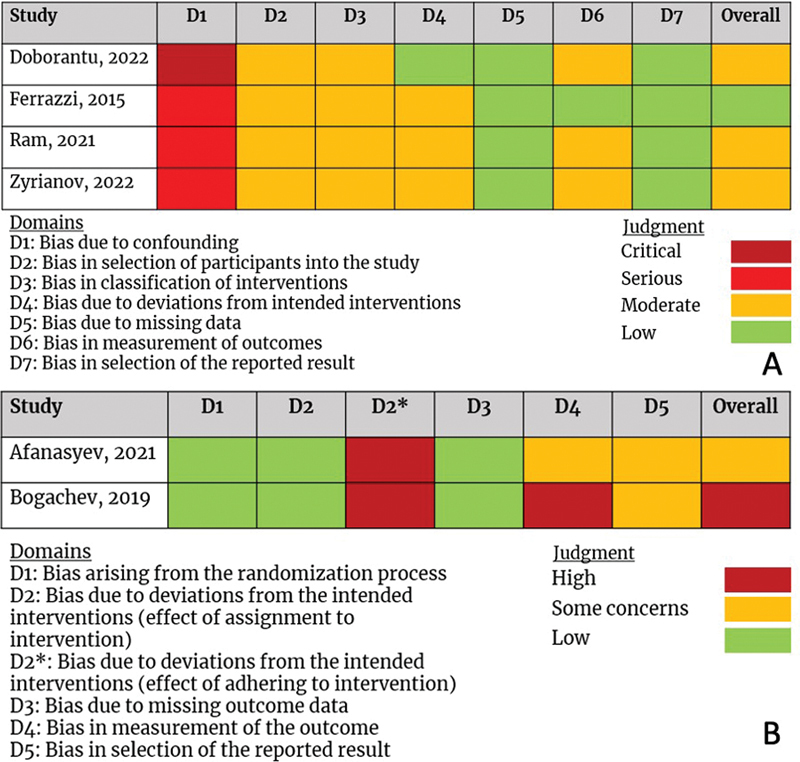
Risk-of-bias summary for ( **A** ) ROBINS-1 and ( **B** ) RoB 2. ROBINS-I, Risk of Bias in Non-Randomized Studies of Interventions; RoB 2, revised Cochrane risk-of-bias tool for randomized trials.

Four of the studies were assessed using the ROBINS-I assessment tool. 12 17 18 19 The article of Ferrazzi et al had low RoB, whereas the remaining three studies were judged to show moderate RoB. 12 The two RCTs were assessed using the RoB 2 tool, with an overall score of high RoB with some concerns. 20 21 The more detailed quality assessment can be found in Fig. 2A (ROBINS-I) and Fig. 2B (RoB 2). A sensitivity analysis, including leave-one-out, stratified analysis, and meta-regression, was not conducted due to low heterogeneity in the primary outcome. Publication bias was assessed for postoperative resting LVOT gradient. Funnel plot asymmetry was not present on visual inspection for the postoperative resting LVOT gradient ( Supplementary Fig. S1 , available in the online version only).

### Baseline Demographic Data

All six studies reported baseline demographic data. Data from a maximum of 471 individual patients were available for analysis, varying on behalf of the reported outcome. The number of patients who underwent a surgical myectomy in combination with secondary chordal cutting was the lowest in the study of Afanasyev et al 21 ( *n* = 24) and the highest ( *n* = 161) in the study of Zyrianov et al. 17 The mean resting LVOT gradients were the lowest in the study of Zyrianov et al 17 (70 ± 34) and the highest in the study of Ram et al 19 (92 ± 17). Neither study measured the provoked LVOT gradient. Afanasyev et al and Bogachev-Prokophiev et al measured the SAM in all patients, while the other studies did not measure the SAM. 12 17 18 19 20 21 The proportion of patients with a preoperative MR grade 3 or 4 was the lowest in the article of Ferrazzi et al 12 (23%) and the highest in the articles of Afanasyev et al 21 and, Bogachev-Prokophiev et al 20 (100%). These results are summarized in Table 1 .

### Echocardiographic Outcomes

**Fig. 3:**
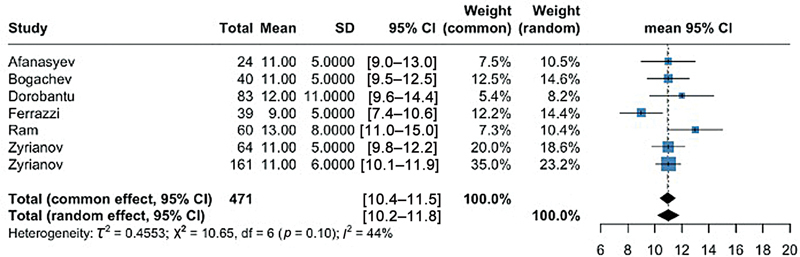
Forest plots representing the pooled mean for postoperative LVOT gradient. The left portions of the figure show the studies analyzed with their corresponding mean or proportion and confidence interval (CI). The right portion of the figure shows a forest plot of the data. CI, confidence interval; LVOT, left ventricle outflow tract obstruction; SD, standard deviation.

The pooled mean postoperative resting LVOT gradient (primary outcome) was 11 mm Hg (95% CI: 10–12; 6 studies, 471 patients; Fig. 3 ). This result was associated with a low heterogeneity ( *I* ^2^ = 44%). Postoperative residual resting LVOT gradient exceeding 30 mm Hg was observed in nine patients (1%). The secondary echocardiographic outcome (i.e., important residual postoperative MR) was observed in 1% of the patients. Echocardiographic outcomes are summarized in Table 2 .

**Table 2:** Echocardiographic outcome at discharge

Study	*N*	Follow-up period	LVOT gradient at rest (mm Hg)	LVOT gradient >30 at rest (mm Hg)	SAM	IVSd (mm)	MR grades 0–1	MR grade 2	MR grades 3 and 4
Afanasyev et al 21	24	Discharge	11 ± 5	1 (4)	2 (8)	18 (15–22)	18 (75)	6 (25)	0 (0)
Bogachev-Prokophiev et al 20	40	Directly postoperative	8 ± 2	0 (0)	2 (5)	15 ± 2	–	–	0 (0)
Dorobantu et al 18	83	Discharge	13 ± 11	5 (6)	–	16 ± 3	52 (63)	27 (33)	1 (1)
Ferrazzi et al 12	39	Intraoperative	9 ± 5	0 (0)	–	–	33 (85)	5 (13)	1 (3)
Zyrianov et al (≤19 mm IVSd) 17	64	Postoperative	11 ± 5	3 (2)	–	14 ± 2	55 (86)	8 (13)	1 (2)
Zyrianov et al (≥20 mm IVSd) 17	161	Postoperative	11 ± 6	0 (0)	–	17 ± 4	139 (86)	19 (12)	3 (2)
Ram et al 19	60	Discharge	13 ± 8	0 (0)	–	13 ± 3	–	–	1 (2)

Abbreviations: IVSd, interventricular septum thickness in diastole; LVOT, left ventricular outflow tract; MR, mitral regurgitation; N, number of patients; SAM, systolic anterior motion; –, data not reported.

Note: Values are mean ± standard deviation (SD), median (interquartile range), or *n* (%).

### Surgical Techniques and Operative Data

In all studies, a transverse aortotomy approach was used for surgical myectomy and secondary chordal cutting. All patients underwent surgical myectomy with additional secondary chordal cutting as described by Ferrazzi et al. 12 The number of secondary chordal removed was described by Dorobantu et al 18 (median: 6; range: 2–13), Bogachev-Prokophiev et al 20 (no median; range: 2–6), and Ferrazzi et al 12 (median: 3; range: 1–8). The aortic cross-clamp (ACC) and cardiopulmonary bypass (CPB) times were presented by Ram et al 19 (43 and 63 minutes), Bogachev-Prokophiev et al 20 (45 and 62 minutes), and Afanasyev et al 21 (53 and 76 minutes). Additionally, 16 patients (6%) underwent concomitant coronary artery bypass grafting (CABG), 1 patient (0.4%) underwent concomitant aortic valve replacement (AVR), and 1 patient (0.4%) underwent concomitant tricuspid valve replacement (TVR). Additionally, 15 patients (6%) underwent concomitant MAZE or pulmonary vein isolation (PVI). In total, four patients (3%; derived from 3 studies including a total of 124 patients) required a second CPB. One case required a second CPB due to a residual significant resting LVOT gradient (33 mm Hg) in combination with SAM-mediated moderate MR necessitating a more extended surgical myectomy. The second patient who required a second CPB was due to residual MR, LVOTO, and SAM, necessitating a more extended surgical myectomy. The remaining two patients required a second CPB for unclear reasons. Procedural characteristics are summarized in Table 3 .

**Table 3:** Intraoperative and 30-day outcomes

Study	*N*	ACC	CPB	Second CPB	VSD	Permanent pacemaker	AF	In-hospital mortality
Afanasyev et al 21	24	53 (21)	76 (13)	1 (4)	0	0	–	0
Bogachev-Prokophiev et al 20	40	45 (14)	62 (15)	1 (3)	1 (3)	2 (6)	–	0
Dorobantu et al 18	83	–	–	–	0	8 (10)	13 (16)	1 (1)
Ferrazzi et al 12	39	–	–	–	0	–	6 (15)	0
Zyrianov et al (≤19mm IVSd) 17	64	–	–	–	0	–	5 (8)	–
Zyrianov et al (≥20 mm IVSd) 17	161	–	–	–	0	–	10 (6)	1 (0.4)
Ram et al 19	60	33 (29–54)	52 (42–65)	2 (3)	–	5 (8)	0	0

Abbreviations: ACC, aorta cross-clamping time in minutes; CPB, cardiopulmonary bypass times in minutes; AF, atrial fibrillation; *N* , number of patients; VSD, ventricular septal defect; –, data not reported.

Note: Values are mean ± standard deviation (SD), median (interquartile range), or *n* (%).

### Complications

The 30-day postoperative permanent pacemaker implantation incidence was 7%. The in-hospital mortality incidence was 0.4%, corresponding to one deceased patient in the study of Dorobantu et al 18 and one patient in the study of Zyrianov et al. 17 The 1-year follow-up mortality incidence was 0.6%, corresponding to one patient who died after hospital discharge and two in-hospital mortality patients as described by Ram et al. 19

## Discussion

This review summarizes the clinical outcomes of the combination of surgical myectomy and secondary chordal cutting in symptomatic HOCM patients. The main findings of the present systematic review and meta-analysis suggest that the combination of surgical myectomy and secondary chordal cutting effectively relieved LVOTO in symptomatic HOCM patients (11 mm Hg; 95% CI: 10–12). The heterogeneity was low ( *I* ^2^ = 44%). Moreover, the presence of either MR grade 3 or 4 was significantly reduced in all articles. The highest proportion of either MR grade 3 or 4 postoperatively was in the study of Ferrazzi et al (1%). 12 Furthermore, our review shows excellent 1-year survival after surgical myectomy in combination with secondary chordal cutting with only 0.6% mortality.

In the past decades, the role of mitral valve repair in addition to surgical myectomy has remained controversial. However, the 2023 ESC guidelines for the management of cardiomyopathies stated that either mitral valve repair or mitral valve replacement should be considered in symptomatic patients with a maximum LVOTO gradient ≥50 mm Hg and moderate to severe MR that cannot be corrected by septal reduction therapy alone. 1 Nevertheless, details regarding the specific types of structural abnormalities that could prohibit adequate correction with surgical myectomy alone and the recommended techniques for addressing these are not provided. 8 HCM patients exhibit distinct phenotypic features of the mitral valve apparatus. These include valvular abnormalities (elongated mitral valve leaflets, increased mitral tenting volume, and smaller coaptation–septal distance), papillary muscle abnormalities (increased number of papillary muscles and abnormal papillary muscle insertion), and chordal apparatus (shortened and fibrotic chordae tendineae). 4 Although the surgical importance for some of these phenotypic features is arguable, it has been previously demonstrated that, specifically, the secondary mitral chords may play a substantial role in LVOT obstruction in HOCM, as they can contribute to SAM by tethering the anterior mitral leaflet toward the LVOT. 4 5 The addition of secondary chordal cutting to surgical myectomy could therefore offer multiple benefits in HOCM patients with SAM and MR, as it alters the coaptation point of the mitral valve, ensuring LVOT widening and SAM reduction and may thus prevent the need for mitral valve replacement. 4 12 Although the incidence of secondary anterior mitral leaflet strut chords is not well established, some patients with these secondary anterior mitral valve struts may benefit from secondary mitral chordal cutting.

Even though this study does not compare the results of surgical myectomy plus additional secondary chordal cutting with isolated surgical myectomy, the present systematic review and meta-analysis showed that a good outcome in terms of LVOT gradient can be achieved by surgical myectomy with secondary chordal cutting, with a significant reduction in the presence of MR grades 3 and 4, while procedural times remain relatively limited as reflected by the mean ACC and CPB times. The findings of the present study are broadly in line with the published literature on patients who underwent isolated surgical myectomy and additional mitral valve surgery. 22 23 24 25 Here, the procedure also effectively achieved a reduction in resting LVOTO mm Hg by more than 90% with an operative mortality rate of approximately 1%. The studies by Bogachev-Prokophiev et al 20 and Ram et al 19 included control groups undergoing isolated surgical myectomy. Bogachev-Prokophiev et al conducted an RCT, while Ram et al conducted an observational study, which is inherently prone to selection bias. Both studies reported significantly lower resting LVOT gradients and a reduction in moderate or more than moderate MR after the addition of concomitant secondary chordal cutting. 19 20

Although a recent study by Nguyen et al previously demonstrated that excellent outcomes can be achieved after surgical myectomy alone in all patients regardless of basal septal thickness, 26 some of the included studies have reported the outcomes according to the degree of septal thickness. Zyrianov et al 17 divided participants into two subgroups based on a septal thickness of ≤19 and ≥20 mm, and Ferrazzi et al 12 only included participants with a mild septal thickness (≤19 mm). In contrast to these two articles, the other four included articles did not make any distinction based on septal thickness. Importantly, the mean interventricular septum thickness in diastole (IVSd) was more than 24 mm in most articles, indicating the presence of more than mild septal thickness in most patients. 17 18 19 20 21

## Limitations

Some limitations should be considered when interpreting the summarized data. First, the study has no control group. Therefore, the evidence regarding the additive benefit of adding secondary chordal cutting remains unclear. Second, although all studies that reported results of concomitant mitral valve procedures other than secondary chordal cutting were excluded, some heterogeneity in other concomitant procedures remained. Specifically, Dorobantu et al 18 and Ram et al 19 did not exclude patients with concomitant surgery (i.e., concomitant CABG, AVR, TVR, or atrial fibrillation ablation) other than secondary chordal cutting, which could introduce confounding by indication. This might have led to an overestimation of procedural times and complication rates including mortality. Moreover, Ferrazzi et al 12 and Bogachev-Prokophiev et al 20 excluded patients with preoperative atrial fibrillation, a factor that might have also introduced confounding by indication. Third, all studies involved relatively small sample sizes, and individual patient-level analysis was not conducted. Fourth, some detailed echocardiographic and clinical data were missing. For example, provoked LVOT gradient was not measured in any study. The obstruction is dynamic in most HOCM patients and therefore any latent obstruction might persist. Preoperative and postoperative SAM was only reported in two articles. Although SAM was initially included as a secondary endpoint, no further analysis was possible. The presence of a right bundle branch block was not presented in any article.

## Conclusion and Future Implications

The cumulative, available, and published evidence as analyzed in this systematic review and meta-analysis shows that the combination of surgical myectomy and secondary chordal cutting can be performed safely and leads to a significant reduction in LVOT gradients and MR, with excellent 1-year survival. Evidence on outcomes in the current literature is limited by inconsistent patient selection criteria, heterogeneous definitions of clinical endpoints, and missing or incomplete data reporting (i.e., provoked LVOT gradient). Ultimately, it is essential to conduct well-planned RCTs to compare HOCM patients undergoing isolated surgical myectomy with HOCM patients undergoing surgical myectomy in combination with secondary chordal cuttings to assess the additive benefit of additional secondary chordal cuttings. Our analysis highlights the potential value of the combination of surgical myectomy and secondary chordal cutting in patients with HOCM, especially regarding further reduction of LVOT gradients and SAM-mediated MR.

## Potential Clinical Implications

- Secondary chordal cutting with surgical myectomy can be performed safely.
- In certain HOCM patients, secondary chordal cutting has the potential value to further reduce SAM, MR, and LVOT gradients.
- Careful assessment of preoperative imaging modalities, particularly transthoracic echocardiography and magnetic resonance imaging, along with perioperative visualization, is crucial for detecting secondary anterior mitral valve leaflet strut chordae.
- Awareness of secondary anterior mitral valve leaflet strut chordae must be raised among physicians when preparing for surgical myectomy in patients with HOCM.
