# Supplementary material for: Echocardiographic and Clinical Outcomes of Concomitant Secondary Chordal Cutting to Surgical Myectomy in Hypertrophic Obstructive Cardiomyopathy: A Systematic Review and Meta-analysis
Source: Thorac Cardiovasc Surg. 2024 Nov 18;73(7):519–28. doi: 10.1055/a-2434-7627 (PMC12503925; doi:10.1055/a-2434-7627)
Supplement: Supplementary file 1 — Supplementary Material [file 10-1055-a-2434-7627-s0820247314r.pdf]

Supplementary Material

Supplementary Search Strings S1

First Search Strings

Syntax Pubmed 1.0

("Cardiomyopathy, Hypertrophic"[Mesh] OR "Hypertrophic Obstructive Cardiomyopath*"[tiab] OR "Hypertrophic Cardiomyopath*"[tiab] OR "Ventricular Hypertroph*"[tiab] OR "Septal Hypertroph*"[tiab] OR "Hypertrophic Subaortic Stenosis"[tiab]) AND (("Heart Septum/surgery"[Mesh:NoExp] OR "Ventricular Septum/surgery"[Mesh] OR "morrow"[tiab] OR "myectom*"[tiab] OR "myomyectom*"[tiab] OR (("Heart Septum"[Mesh:NoExp] OR "Ventricular Septum"[Mesh] OR "septum"[tiab] OR "septal"[tiab]) AND ("resection*"[tiab] OR "surgery"[tiab] OR "surgeries"[tiab] OR "surgical"[tiab]))))

2,100 results, March 18, 2023

Syntax Embase

('hypertrophic cardiomyopathy'/exp OR 'Hypertrophic Obstructive Cardiomyopath*':ti,ab,kw OR 'Hypertrophic Cardiomyopath*':ti,ab,kw OR 'Ventricular Hypertroph*':ti,ab,kw OR 'Septal Hypertroph*':ti,ab,kw OR 'Hypertrophic Subaortic Stenosis':ti,ab,kw OR 'HOCM':ti OR 'HCM':ti) AND (('morrow':ti,ab,kw OR 'myectom*':ti,ab,kw OR 'myomyectom*':ti,ab,kw OR 'VSM':ti OR 'SM':ti OR (('heart septum'/de OR 'interventricular septum'/de OR 'septum':ti,ab,kw OR 'septal':ti,ab,kw) AND ('resection*':ti,ab,kw OR 'surgery':ti,ab,kw OR 'surgeries':ti,ab,kw OR 'surgical':ti,ab,kw))))

2,940 results, March 18, 2023

Second search strings

Syntax PubMed 2.0

("Cardiomyopathy, Hypertrophic"[Mesh] OR "Hypertrophic Obstructive Cardiomyopath*"[tiab] OR "Hypertrophic Cardiomyopath*"[tiab] OR "Ventricular Hypertroph*"[tiab] OR "Septal Hypertroph*"[tiab] OR "Hypertrophic Subaortic Stenosis"[tiab]) AND (("Heart Septum/surgery"[Mesh:NoExp] OR "Ventricular Septum/surgery"[Mesh] OR "morrow"[tiab] OR "myectom*"[tiab] OR "myomyectom*"[tiab] OR (("Heart Septum"[Mesh:NoExp] OR "Ventricular Septum"[Mesh] OR "septum"[tiab] OR "septal"[tiab]) AND ("resection*"[tiab] OR "surgery"[tiab] OR "surgeries"[tiab] OR "surgical"[tiab]))))

Publication date from March 18, 2023

153 results, April 12, 2024

Syntax Embase

('hypertrophic cardiomyopathy'/exp OR 'Hypertrophic Obstructive Cardiomyopath*':ti,ab,kw OR 'Hypertrophic Cardiomyopath*':ti,ab,kw OR 'Ventricular Hypertroph*':ti,ab,kw OR 'Septal Hypertroph*':ti,ab,kw OR 'Hypertrophic Subaortic Stenosis':ti,ab,kw OR 'HOCM':ti OR 'HCM':ti) AND (('morrow':ti,ab,kw OR 'myectom*':ti,ab,kw OR 'myomyectom*':ti,ab,kw OR 'VSM':ti OR 'SM':ti OR (('heart septum'/de OR 'interventricular septum'/de OR 'septum':ti,ab,kw OR 'septal':ti,ab,kw) AND ('resection*':ti,ab,kw OR 'surgery':ti,ab,kw OR 'surgeries':ti,ab,kw OR 'surgical':ti,ab,kw))))

259 results, April 12, 2024

Supplementary Methods S2

Data Selection

The study population under review comprised adult hypertrophic obstructive cardiomyopathy (HOCM) patients aged ≥18 years who underwent surgical septal myectomy with additional secondary chordal cutting. Randomized controlled trials (RCTs) and nonrandomized studies, including case control studies and cohort studies or case series with a minimum of five reported patients, were eligible for inclusion. Articles were retrieved for review if they were written in the English language only and only involving human subjects. Studies were also excluded if the article was a review, conference abstract, editorial, or case report. When trials, registries, or institutions published duplicate studies with extended lengths of follow-up or larger study populations, the most updated and complete study was included.

Data Extraction

The following data were extracted from all included studies: main author's last name, year of publication, number of participants, study design, patient characteristics (age, gender, history of atrial fibrillation, and New York Heart Association [NYHA] class); intraoperative data (mitral valve repair technique, aortic cross-clamp [ACC] time, cardiopulmonary bypass [CPB] time, concomitant surgery, and eventual second ACC time); pre- and postoperative echocardiographic parameters (left ventricular ejection fraction [LVEF], SAM, interventricular septal thickness in diastole [IVSd], resting left ventricular outflow tract [LVOT] pressure gradient, and mitral regurgitation [MR] grade); complications (ventricular septal defect [VSD], new permanent pacemaker, new-onset atrial fibrillation, and in-hospital and 1-year all-cause mortality) and duration of follow-up. MR was categorized into five grades, according to the recommendation of the European Association of Cardiovascular Imaging (EACVI)[1]: none or trace = grade 0; mild = grade 1; mild to moderate = grade 2; moderate to severe = grade 3; severe = grade 4.

 © 2024. The Author(s).

Supplementary Table S1 Detailed reasons to exclude articles

Study	Reasons exclusion	Excluded
Binaco et al[2]	Case report	Case report
Ji et al[3]	68% of the patients underwent surgical septal myectomy in combination with secondary chordal cutting. The other patients (32%) underwent other concomitant mitral valve procedures	Pooled mitral valve
Margonato et al[4]	This study has overlap with the study of Zyrianov et al [5] Klik of tik om tekst in te voeren. This was a conferential abstract	Overlapping cohorts
Liu et al[5]	75% of the patients underwent surgical septal myectomy in combination with secondary chordal cutting. The other patients (25%) underwent other concomitant mitral valve procedures	Pooled mitral valve
Raffa et al[6]	Patients also underwent mitral valve replacement or other concomitant procedures to the mitral valve	Pooled mitral valve
Hayashi et al[7]	Patients were initially stratified for mitral regurgitation. Only in supplemental stratified for chordal cuttings. We cannot use these data in our systematic review and meta-analysis	Pooled baseline characteristics

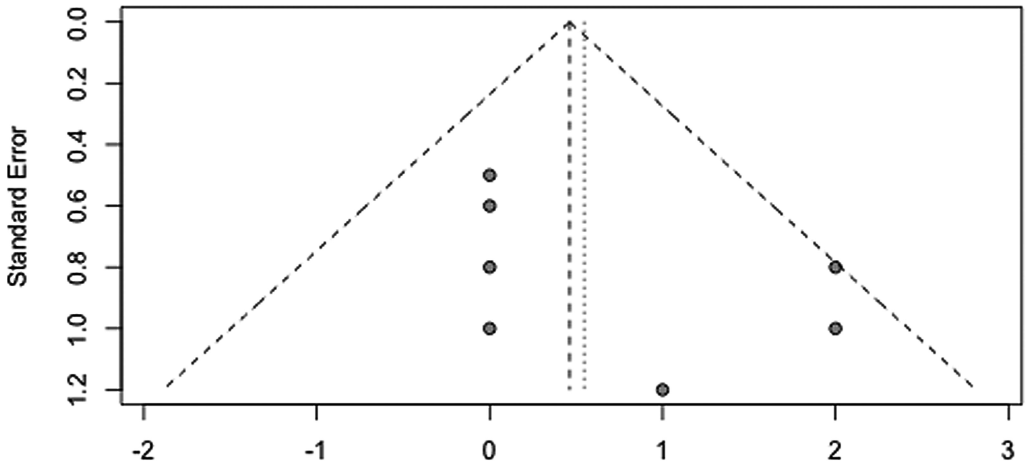

Supplementary Fig. S1 Funnel plot
Funnel plot illustrating the distribution of mean difference plotted against their standard error, for the primary outcome (postoperative resting left ventricular outflow tract [LVOT] gradient). The funnel plot suggests symmetry, which does not suggest any publication bias or other sources of heterogeneity within the included studies.

 © 2024. The Author(s).

References

1 Lancellotti P, Pibarot P, Chambers J, et al. Multi-modality imaging assessment of native valvular regurgitation: an EACVI and ESC council of valvular heart disease position paper. Eur Heart J Cardiovasc Imaging 2022;23:e171–e232

2 Binaco I, Spirito P, Poggio D, Casati V, Grillo M, Ferrazzi P. Transaortic mitral valve secondary chordal cutting in patients with obstructive hypertrophic cardiomyopathy and mild septal hypertrophy. Ann Cardiothorac Surg 2017;6:426–428

3 Ji Q, Wang Y, Yang Y, et al. Characteristics and surgical results of patients with hypertrophic obstructive cardiomyopathy without intrinsic mitral valve diseases undergoing mitral subvalvular procedures during myectomy. Cardiol Res Pract 2020;2020:1–9

4 Margonato D, Abete R, Zyrianov A, et al. Systematic cutting of selected secondary mitral valve chordae, in association with a shallow myectomy, in obstructive hypertrophic cardiomyopathy: impact on mitral valve function and patient management. Eur Heart J 2020;41:ehaa946.2076

5 Liu F, Wang Y, Yang Y, et al. Myectomy with and without mitral subvalvular repair in patients with hypertrophic obstructive cardiomyopathy with grade 3 to 4+ mitral regurgitation without intrinsic mitral valve disease: a retrospective observational study. Rev Cardiovasc Med 2022;23:279

6 Raffa GM, Franca ELa, Lachina C, et al. Septal thickness does not impact outcome after hypertrophic obstructive cardiomyopathy surgery (septal myectomy and subvalvular mitral apparatus remodeling): a 15-years of experience. Front Cardiovasc Med 2022;9:1–10

7 Hayashi H, Singh SK, Hahn RT, et al. Mitral regurgitation mechanisms related to systolic anterior motion in hypertrophic cardiomyopathy. J Thorac Dis 2024;16:26–39

 © 2024. The Author(s).
